# Autologous-cell-derived, tissue-engineered cartilage for repairing articular cartilage lesions in the knee: study protocol for a randomized controlled trial

**DOI:** 10.1186/s13063-017-2251-6

**Published:** 2017-11-06

**Authors:** Ning Ma, Hongxia Wang, Xian Xu, Yiqun Wan, Yufeng Liu, Mingjie Wang, Wen Yu, Yongjing Dai, Jiang Peng, Quanyi Guo, Changlong Yu, Shibi Lu

**Affiliations:** Institute of Orthopaedics, Chinese PLA General Hospital, No. 28 Fuxing Road, Beijing, 100853 China

**Keywords:** Tissue-engineered cartilage, Microfracture, Cartilage injury, Autologous chondrocytes, Acellular cartilage matrix, Bionic scaffold, Randomized controlled, Clinical trials

## Abstract

**Background:**

Spontaneous recovery from articular cartilage injury is difficult, and the ongoing progression of disease can eventually lead to osteoarthritis. Currently, there is no effective non-surgical treatment for articular cartilage injury. Arthroscopic debridement and microfracture surgery are performed for fibrocartilage repair. But fibrocartilage is different from normal articular cartilage, and functional recovery is not satisfactory. Therefore, it is necessary to develop more effective techniques for articular cartilage repair. Progress in material science, cell biology, biomechanics, and bioreactor technology has allowed the development of biomimetic, tissue-engineered osteochondral composites that have shown potential for the repair of damaged cartilage. We prepared biomimetic, tissue-engineered cartilage scaffolds optimized for biochemical composition and structural characteristics. Based on the experience of our pre-clinical studies on animals, a human articular cartilage acellular matrix scaffold was prepared and is in clinical use. The combination of autologous chondrocytes and scaffolds has shown satisfactory results in repairing cartilage defects in preliminary experiments.

**Methods:**

This is a prospective randomized controlled trial. One hundred patients with full-thickness cartilage injury of the knee will be randomly divided into two groups to receive treatment with either tissue-engineered cartilage constructed using biomimetic cartilage extracellular-matrix-oriented scaffolds combined with autologous chondrocytes, or arthroscopic debridement and microfracture surgery. There will be five visiting time points: at baseline, then at 3, 6, 12, and 18 months postoperatively. The primary outcome will be therapeutic efficacy as assessed by the Lysholm score at 12 months postoperatively. The secondary outcomes will be the International Knee Documentation Committee score, Visual Analog Scale score, and cartilage injury and repair as assessed by magnetic resonance imaging as well as the incidence of postoperative adverse events.

**Discussion:**

This trial will attempt to verify the use of tissue-engineered cartilage constructed using autologous chondrocytes combined with allogeneic, acellular cartilage matrix for the repair of cartilage defects, thereby providing favorable evidence for its use in clinical practice.

**Trial registration:**

ClinicalTrials.gov, identifier: NCT02770209. Registered on 11 May 2016.

**Electronic supplementary material:**

The online version of this article (doi:10.1186/s13063-017-2251-6) contains supplementary material, which is available to authorized users.

## Background

Injured articular cartilage has limited capacity for self repair. Without timely, early, and effective treatment, damage to the articular cartilage progressively worsens, resulting in joint swelling, pain, and dysfunction. The patient ultimately develops osteoarthritis and may require artificial joint replacement. Clinical therapy for cartilage damage includes microfracture surgery and autologous osteochondral transplantation. However, the microfracture technique is limited to small-scale damage, and autologous osteochondral transplantation is hindered by limited supply. With advances in material science, cell biology, biomechanics, and bioreactor technology, the new generation of biomimetic, tissue-engineered osteochondral composites display great potential for the repair of cartilage damage [1, 2].

Currently, in cartilage tissue engineering, seed cells are derived from autologous or allogeneic chondrocytes, mesenchymal stem cells, embryonic stem cells, or pluripotent stem cells [3, 4]. Increasing evidence indicates that bone marrow mesenchymal stem cells can be induced to differentiate into chondrocytes, and these cells have been successfully used in the treatment of large-size bone defects, cartilage lesions, and spinal cord injury [5, 6]. The quality and quantity of bone mesenchymal stem cells gradually decreases with age, especially in patients with degenerative diseases [7]. Adipose stem cells and umbilical cord mesenchymal stem cells are abundant and have similar characteristics to bone mesenchymal stem cells, and both of these cell types can be induced to differentiate into chondrocytes [8, 9]. Adipose stem cells and umbilical cord mesenchymal stem cells have been used to repair cartilage defects, but the findings are still preliminary, and these cells cannot be harvested or cultured in large quantities. Furthermore, the use of embryonic stem cells is complicated by ethical considerations. As a consequence, autologous chondrocytes are optimal seed cells for cartilage tissue engineering [10].

The transplantation of autologous chondrocytes in combination with tissue-engineered cartilage scaffolds to repair cartilage damage requires researchers to focus on two major issues, namely (1) the in vitro amplification of chondrocytes and (2) the preparation of biocompatible chondrocyte scaffolds [11]. The preparation of chondrocyte scaffolds requires advanced technique, and currently, only the Institute of Orthopedics at the Chinese PLA General Hospital has the capacity to produce acellular cartilage; there is no other source of tissue-engineered cartilage scaffolds in China.

### Preliminary experiments

**Fig. 1:**
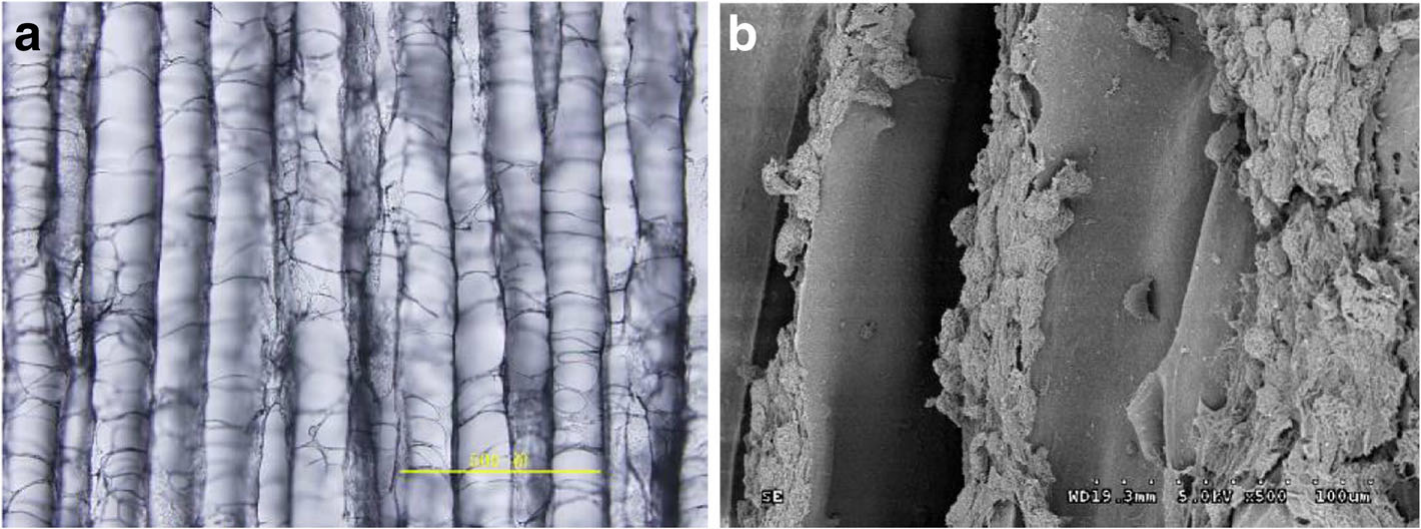
Allogeneic, acellular cartilage scaffolds. Scaffold pores exhibit a parallel arrangement, which is conducive to the growth of cells along the pores. Cells are arranged in a column during proliferation. Cells display a columnar arrangement in line with the normal structural arrangement of articular cartilage, which is conducive to the formation of normal articular cartilage. The pore structure of the oriented scaffold also allows the metabolism of cell nutrition. **a** Light microscope × 100. **b** Scanning electron microscope × 100

**Fig. 2:**
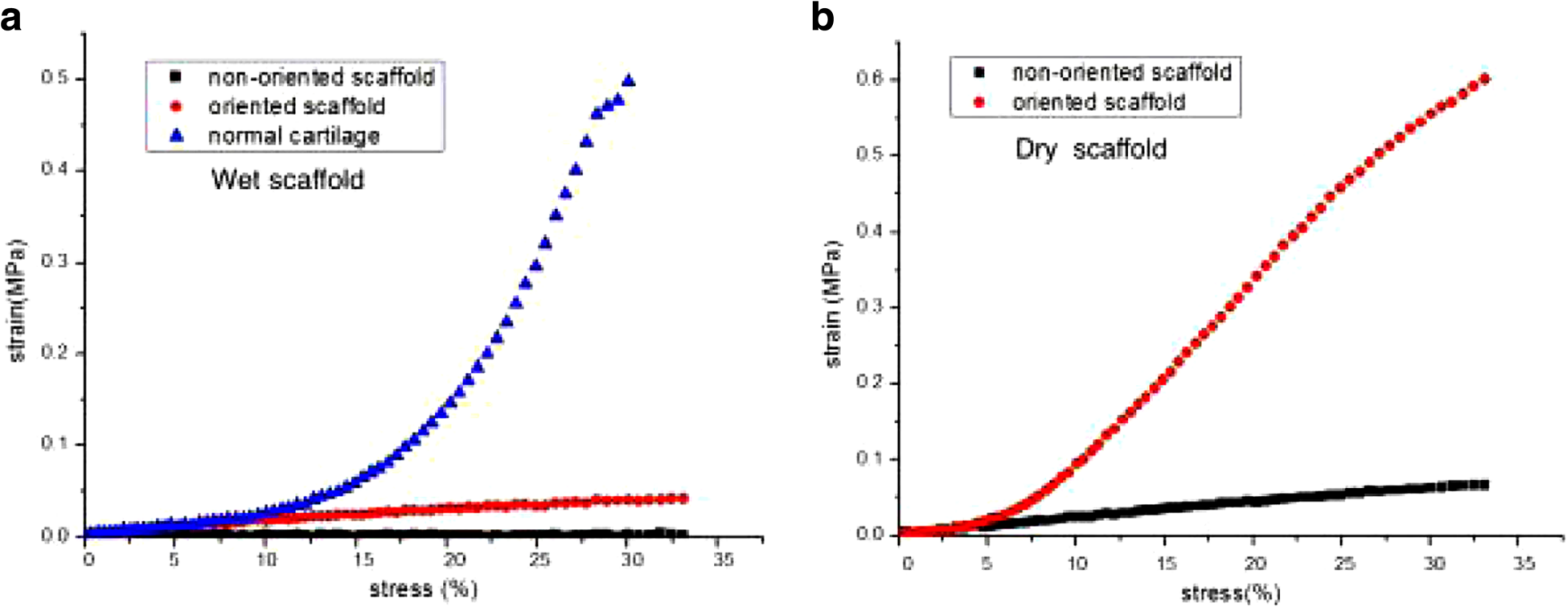
Compressive stress-strain curves. Comparison of biomechanical properties of oriented and non-oriented scaffolds of allogenic, acellular cartilage. **a** Comparison of compressive stress between the oriented and non-oriented scaffolds in a wet condition. **b** Comparison of compressive stress between the oriented and non-oriented scaffolds in a dry condition

A proprietary allogeneic, acellular, cartilage-oriented scaffold was successfully created by the Cartilage Tissue Engineering Research Group, Institute of Orthopedics, Chinese PLA General Hospital (with intellectual property rights). The innovative scaffold simulates the composition and spatial structural characteristics of normal cartilage. The preparation methods are as follows: articular cartilage is pulverized to obtain natural cartilage extracellular matrix, which is identical in biochemical composition to extracellular matrix of natural articular cartilage. Then, a porous sponge-like scaffold is prepared using a freeze-drying technique [12]. In vitro experiments and large-animal, articular cartilage injury repair experiments have produced good results [13–15]. Using this material, our research group prepared biomimetic cartilage tissue-engineered scaffolds, which mimic the structural characteristics of natural articular cartilage extracellular matrix. This allogeneic, acellular cartilage scaffold has the following characteristics: (1) it is derived from allogeneic cartilage, and the extracellular matrix remains intact after allografting, helping to maintain the numerous components of normal cartilage, particularly type II collagen and proteoglycans, resulting in enhanced repair. Cartilage scaffolds used outside of China are mainly composed of types I and III collagen or hyaluronic acid, and vary greatly from natural cartilage components [16]. The original cartilage structure is difficult to reproduce with these types of scaffolds, and fibrous cartilage may affect treatment outcome; (2) the biomimetic scaffold has a similar three-dimensional structure to that of normal articular cartilage, which is the oriented scaffold structure. The scaffold imitates the orientation of normal cartilage cells, which are arranged perpendicular to the surface, and provides a paratactic columnar structure that contributes to the columnar arrangement of cells (Fig. 1) [15]. This structure in combination with type II collagen and proteoglycans derived from normal articular cartilage results in a scaffold structure that is extremely close to that of normal joint cartilage. Consequently, the repaired cartilage will have normal structure and function; (3) the oriented scaffold has a good biomechanical property. Its compressive stress is better than the non-oriented scaffold in wet and dry conditions (Fig. 2); and (4) the oriented scaffold has good biocompatibility. Preliminary experiments have investigated the immune responses of the oriented scaffold of heterogeneous (porcine) and conspecific (rabbit) acellular cartilage. After the oriented scaffold had been implanted into the rabbit, its immune responses were observed from the aspects of cellular immunity and humoral immunity. Results suggested that its immunogenicity was low. Thus, it is verified that the oriented scaffold of acellular cartilage has good biocompatibility [17].

### Theoretical evidence

Cartilage has limited regenerative capacity and can only be repaired using autologous, allogeneic cartilage or artificial substitute materials after injury. However, these repair methods have limited effectiveness. Autologous chondrocyte transplantation may have significant side effects, while allogeneic cartilage or artificial substitute materials have poor biocompatibility. Tissue-engineered cartilage may provide an alternative means of repairing damaged cartilage. After autologous chondrocytes cultured in vitro are combined with a biomimetic cartilage scaffold, the tissue-engineered cartilage is constructed in vitro and transplanted into the site of injury to repair the cartilage. This strategy may restore articular cartilage function, providing a novel method for the treatment of cartilage defects.

### Reason for establishing a control group

Microfracture is an arthroscopic bone marrow stimulation technique first applied in clinical practice by Steadman and Rodrigo in 1985. During microfracture surgery, damaged articular cartilage is cleaned until the edge of the normal articular cartilage and subchondral bone marrow cells, chondrocytes, and bone cells penetrate into the damaged area through a hole made using an awl on the surface of the exposed subchondral bone. Blood clots seeping out of the hole adhere to surrounding normal cartilage tissues forming a fibrous cartilage repair defect area responsible for joint function recovery [18]. Arthroscopic microfracture is the most widely used method for the surgical repair of articular cartilage injuries because it is a simple operation with satisfactory clinical efficacy [19]. Here, microfracture surgery will be used as a control for the repair of articular cartilage injury.

## Methods/design

### Study objectives

We hypothesized that autologous-cell-derived, tissue-engineered cartilage is superior to microfractures in the treatment of knee cartilage injury.

The main objective of the study is to evaluate the functional and symptomatic recovery in patients with knee joint cartilage injury undergoing autologous-cell-derived cartilage treatment and to compare it with microfracture surgery, based on Lysholm scores at 12 months postoperatively as the primary outcome measure.

The secondary objective of the study is to (1) compare the effectiveness of the two repair methods on knee function recovery based on International Knee Documentation Committee (IKDC) scores, (2) compare the effectiveness of the two repair methods on pain relief, (3) assess cartilage regeneration by magnetic resonance imaging (MRI), and (4) compare the safety of the two repair methods for knee cartilage repair.

### Study design

**Fig. 3:**
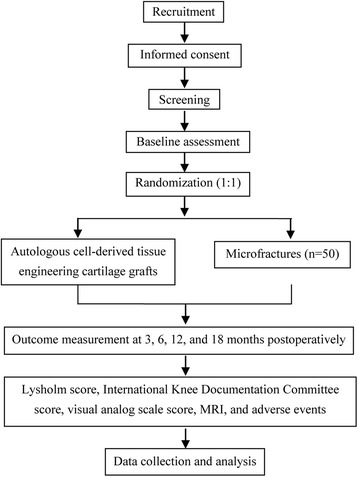
Flow chart of the trial

**Fig. 4:**
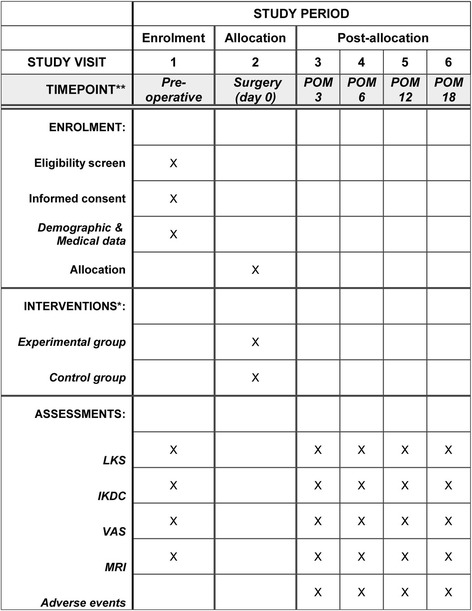
Standard Protocol Items: Recommendations for Interventional Trials (SPIRIT) Figure

This prospective, single-blind, randomized controlled clinical trial will be conducted at the Institute of Orthopaedics, Chinese PLA General Hospital, China. The study design is compliant with the Standard Protocol Items: Recommendations for Interventional Trials (SPIRIT) Statement [20] (see Additional file 1). After providing informed consent, the potential patients with full-thickness cartilage injury of the knee joint will be screened according to the inclusion and exclusion criteria, and finally, 100 eligible patients will be randomly assigned to receive autologous-cell-derived tissue-engineered cartilage grafts (*n* = 50) or microfractures (*n* = 50). The primary outcome is therapeutic efficacy as assessed by the Lysholm score at 12 months postoperatively. The secondary outcomes are the IKDC score, Visual Analog Scale (VAS) score, and cartilage injury and repair as assessed by MRI as well as the incidence of postoperative adverse events (see the trial flow chart in Fig. 3). The Standard Protocol Items: Recommendations for Interventional Trials (SPIRIT) Figure is detailed in Fig. 4.

### Inclusion criteria

Patients meeting all of the following criteria will be considered for admission to the trial:

- ○ Patients with full-thickness cartilage injury of the knee joints
- ○ Patients with normal joint movement and a stable joint (without injury or less than one third excision of the meniscus; normal cruciate ligament, lateral and medial collateral accessory ligament, or normal Q-angle and patellofemoral joint trajectory after transplantation, or corrected to normal by surgery), and without valgus or varus deformity
- ○ Patients aged 18–50 years of age (patients over 50 years of age have poor viability of autologous chondrocytes)
- ○ Patients with focal cartilage defects diagnosed by arthroscopy, Outerbridge III/IV grade, cartilage defect size 1–5 cm^2^, intact articular surface (lower than grade II injury according to the Outerbridge classification), and one or two lesions in the same joint
- ○ Patients and their families who are informed of the treatment and who provide signed informed consent

### Exclusion criteria

Patients presenting with one of the following criteria will not be included in the trial:

- ○ Autoimmune disease (rheumatoid arthritis, chronic kidney disease)
- ○ Hematonosis (thrombocytopenia, leukemia)
- ○ Topical steroid therapy within 3 months
- ○ Bleeding tendency
- ○ Drug addiction (including narcotic, anesthetic, or alcohol addiction)
- ○ Inflammatory joint disease (specific or non-specific arthritis)
- ○ Contagious viral infection
- ○ Metabolic diseases (gout or rheumatism)
- ○ Body Mass Index > 30 kg/m^2^
- ○ Pregnant or lactating women, or planning to become pregnant within 1 year after initial registration
- ○ Psychological mental illness, cannot cope with rehabilitation

### Recruitment

Patients with full-thickness cartilage injury of the knee joint will be recruited from outpatients and inpatients at the PLA General Hospital. Patients who are interested in participation in the trial can contact the sponsor through their attending physicians via telephone, Email, or WeChat. Recruitment information will be issued through hospital websites, social software, and posters.

### Randomization and blinding

A randomization sequence table will be generated using SAS 9.1 software (copyright SAS Institute Inc., Cary, NC, USA) by professional statisticians who will not participate in the trial. Each patient will be randomly assigned a sequence number. The random number will be concealed in sealed envelopes and saved by a researcher who will not participate in the trial. If severe adverse events occur, the researcher will be informed of the need to open the envelope through communication from the principal investigator in situations where code break is warranted. After providing informed consent, eligible patients will be assigned randomly. The final unblinding will be done after data collection.

Surgeons and patients will be aware of the surgical methods used, and evaluators and statisticians will be blinded to group information.

### Interventions

#### Therapeutic approach

##### Interventions in the experimental group

Patients in the experimental group will be treated with the biomimetic cartilage matrix combined with autologous chondrocytes. The entire course of treatment includes two surgeries. The first surgery will be performed to observe articular cartilage damage by arthroscopy and assess treatment potential. If the patient’s condition meets the surgical requirement, we will extract cartilage from the intercondylar fossa non-weight-bearing area during the first surgery. Chondrocytes will be in vitro-amplified and inoculated into the cartilage scaffold at a density of about 1 × 10^7^/mL. The fully prepared seeded scaffold will be implanted into the site of injury during the second surgery.

First surgery (arthroscopic assessment of cartilage damage and harvesting of cartilage seed cells): the scope, location, and size of articular cartilage injury will be assessed with preoperative MRI. Joint ligament and meniscus injury will be evaluated. Mechanical distribution of bony structure around the joint will be evaluated with X-ray examination. Anesthesia will include regional block, spinal anesthesia, or general anesthesia. Patients will be restrained in the supine position during arthroscopy, with their limbs lowered, followed by arthroscopic debridement of the knee joint (including joint lavage, synovectomy, treatment of articular cartilage damage, removal of loose bodies and osteophytes, meniscus surgery, intercondylar fossa and anterior cruciate ligament impingement syndrome treatment).

Assessment and sampling: articular cartilage damage, including the severity, location, size, and depth, will be assessed by arthroscopy. Normal, full-thickness cartilage 100-200 mg (approximately 0.5 cm × 1 cm) will be harvested from the non-weight-bearing area of the femoral condyle or the intercondylar fossa.

Obtaining chondrocytes: the harvested cartilage tissue will be preserved and transported in a 50-mL centrifuge tube containing 8 mL of tissue preservation solution, placed on a test tube rack on top of an ice pack, at 4 °C under sterile conditions. Cartilage tissue will be transferred to the Institute of Orthopedics at the Chinese PLA General Hospital within 12 h. In strict accordance with the requirements and inspection standards of the National Institutes for Food and Drug Control, autologous chondrocytes will be isolated, cultured, expanded, and quality controlled. The culture period will be 3–4 weeks. The cultured cells will be tested for cell viability and secretory function, as well as for bacterial and mycoplasma contamination. Experimental data will be recorded, and figures will be generated. Obtained cartilage seed cells will be frozen in liquid nitrogen for the construction of autologous-cell-derived tissue-engineered cartilage.

Construction of the in vitro tissue-engineered cartilage: after cell culture and expansion, the collected cell suspension will be transferred to a centrifuge tube and centrifuged at 1000 rpm for 10 min to obtain a cell pellet. The supernatant is discarded. The cell pellet will be placed into 10 mL of culture medium, pipetted evenly, and then centrifuged for 10 min and the supernatant discarded. Then, 1 mL of the culture medium will be added to resuspend the chondrocytes followed by inoculation onto a biomimetic cartilage scaffold, forming a bionic cartilage patch. The patch is incubated in an incubator for 30 min before use in surgery.

Second surgery (autologous chondrocyte-constructed, tissue-engineered cartilage for the repair of articular cartilage damage): tissue-engineered cartilage constructed using autologous chondrocytes will be implanted to repair the damaged articular cartilage. Rehabilitation therapy will follow after repair surgery.

Construction of tissue-engineered cartilage in vitro: human chondrocytes will be stored in cryotubes in liquid nitrogen. The cells will be placed in warm water at 40 °C to thaw them within 1 min. The cells will then be transferred into a centrifuge tube and centrifuged for 10 min at 1000 rpm and the supernatant will be discarded. Cells will be resuspended in a 10-mL volume of culture medium, triturated and centrifuged for another 10 min, and the supernatant will be discarded. The cells will be resuspended in fresh culture medium and incubated at 37 °C under 5% CO_2_. Subsequently, the chondrocytes will be seeded onto biomimetic cartilage matrix scaffold to prepare tissue-engineered cartilage.

Eligibility criteria for surgeons: (1) attending physicians with more than 10 years of standing and experience, (2) experience of more than 200 arthroscopic surgeries per year, and (3) having experience and ability to deal with emergencies. Cell culture and construction of tissue-engineered cartilage will be done by a specially trained technologist-in-charge.

##### Interventions in the control group

Patients in the control group will be subjected to arthroscopic debridement and microfracture surgery. Regional block, intraspinal anesthesia, or general anesthesia will be used during surgery. Patients will be restrained in a supine position during arthroscopy, with their affected limb drooped, followed by joint debridement, including joint lavage, synovectomy, treatment of articular cartilage injury, removal of free bodies and osteophytes, meniscus surgery, treatment of intercondylar fossa and anterior cruciate ligament impingement syndrome. After removal of floating cartilage pieces, the joint will be cleaned of calcified bone using a scraper, and tiny fracture holes will be drilled outwardly in a rotational manner with an interval of 3–4 mm at the center of the subchondral bone to ensure the leakage of blood and bone marrow (containing some stem cells) from the holes to form blood clots that differentiate into chondrocytes. After surgery, these chondrocytes will gradually become fibrous cartilage components.

Eligibility criteria for surgeons: (1) attending physicians with more than 10 years of standing and rich experience, (2) experience of more than 200 arthroscopic surgeries per year, and (3) having experience and ability to deal with emergencies.

#### Concomitant care and interventions

Proper rehabilitation and good lifestyle will allow patients to regain their health as soon as possible. The following actions should be avoided to obtain maximum rehabilitation including: sitting on a low bench (about 20 cm in height), using a squat-toilet, strenuous exercise, accidental falls, internal and external rotation of the knee joint, and lying on the affected side. After muscle-strength training, patients will feel mild muscle soreness. The presence of mild muscle soreness and ligament strain or muscle soreness at the second day after exercise is normal and will lead to strengthened muscles and stabilized joints. However, a rest plus ice compress is necessary if the pain lasts for several days, which indicates excessive exercise. If the knee pain is severe and the patient’s activities are severely limited, patients should see a physician immediately.

### MRI determination

#### In vivo evaluation of regenerative cartilage with T2 value

**Fig. 5:**
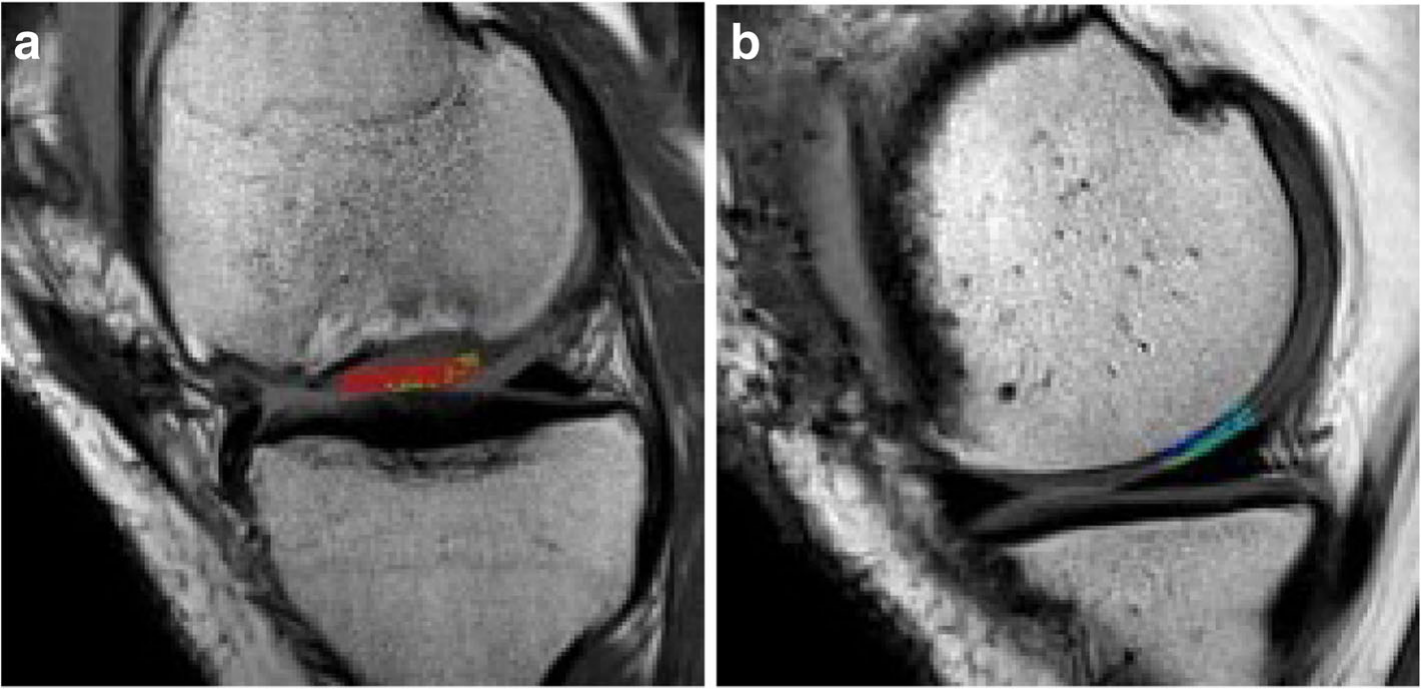
Example of the measurement of T2 values. The regenerative cartilage in this case is located in the medial femoral condyle. **a** Shows the T2 value of regenerative cartilage. **b** Shows the T2 value of normal control cartilage. The color in the region of interest indicates the T2 value

The T2 value is sensitive to the orientation and concentration of collagen, the integrity of the collagen network and water content in cartilage (Fig. 5). To longitudinally evaluate the water content and collagen fiber orientation of cartilage with T2 mapping, MRI examination of T2 mapping will be performed and T2 values of regenerative cartilage will be measured at 3, 6, 12, and 18 months post operation.

T2 mapping is a functional index to evaluate the composition of articular cartilage. The spatial distribution of T2 indicates the water and collagen content and improvement of the direction, which is beneficial for the objective and dynamic monitoring of the regenerated cartilage. The T2 value of the cartilage is an important indicator to measure the structural integrity of collagen fibers, based on which, we can dynamically detect the biochemical properties and maturation process of the repaired tissues.

#### In vivo evaluation of regenerative cartilage with △R1 value

**Fig. 6:**
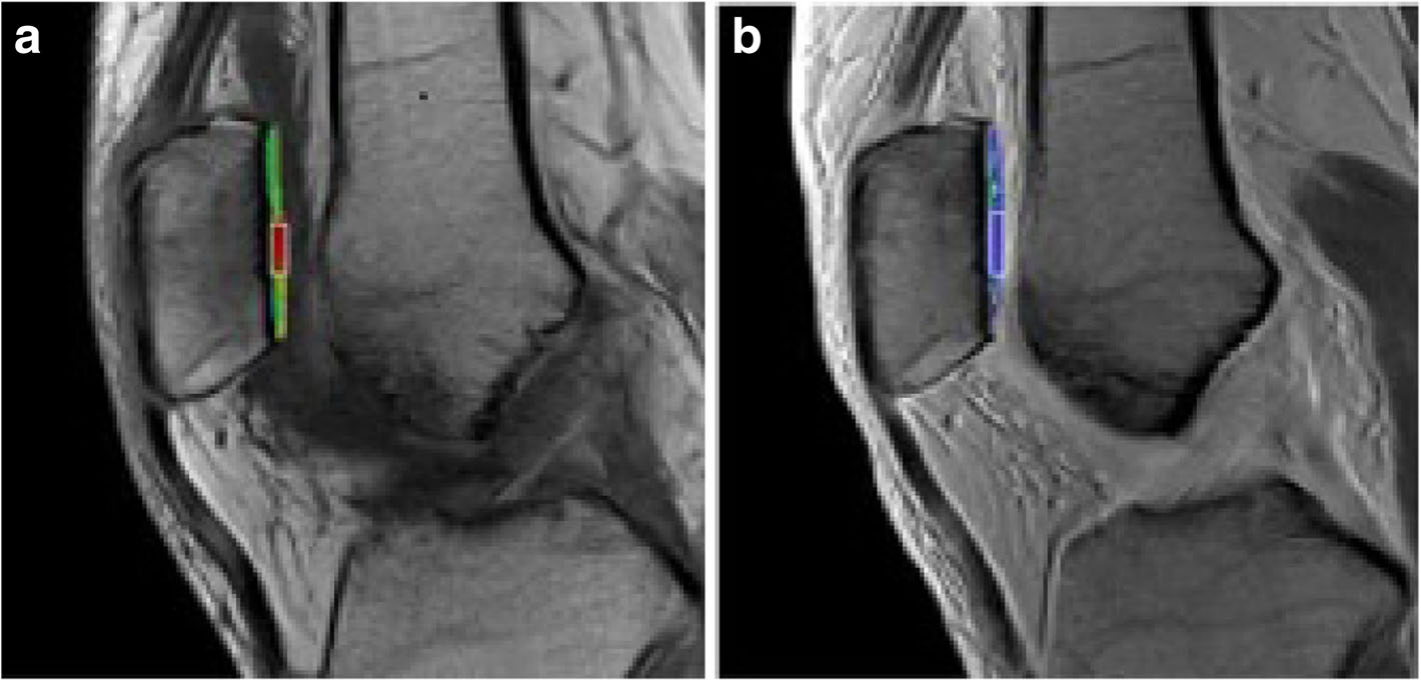
Example of the measurement of T1 values. The regenerative cartilage in this case is located on the patella. **a** Shows the T1 value of regenerative cartilage. **b** Shows the T1 value of normal control cartilage. The color in the region of interest reflects the T1 value

Delayed Gadolinium-enhanced Magnetic Resonance Imaging of Cartilage (dGEMRIC) is an imaging technique that indirectly reflects joint cartilage glycosaminoglycan (GAG) content based on the ion distribution of fixed charge density (FCD) (Fig. 6). GAG molecules with negatively charged hydroxyl and sulfate groups indicate cartilage FCD. Gd-DTPA^2−^, an MRI contrast agent, is also negatively charged, and its distribution in the cartilage is negatively correlated with FCD; therefore, there is a negative correlation between Gd-DTPA^2−^ distribution and GAG content. △*R1* = 1/*T1*post−1/*T1*pre. Higher GAG content is accompanied by gradually reduced △*R1* values, which is used to determine regeneration of the regenerated cartilage [21].

### Outcome measures

#### Primary outcome measure

The Lysholm score for efficacy evaluation will be recorded at 12 months postoperatively. The Lysholm score [22] ranges from 0–100 points and consists of eight dimensions: a score of 80–100 points indicates elimination of all or the main symptoms, basic recovery of joint function, being capable of participating in normal labor and work (excellent); 60–79 points indicates elimination of all or the main symptoms, basic recovery or great improvement in the main function of the joint (good); and 0–59 points indicates no symptom improvement or symptom deterioration (poor). Efficiency = the number of excellent and good cases/the total number of cases × 100%.

#### Secondary outcome measures

- Lysholm score at baseline, 3, 6, and 18 months after operation
- International Knee Documentation Committee (IKDC) score [23] at baseline, 3, 6, 12, and 18 months after operation. The IKDC scores range from 0–100 points and involves symptoms, sport activities, and function, with higher scores reflecting a better condition (knee function and symptoms)
- Visual Analog Scale (VAS) score [24] at baseline, 3, 6, 12, and 18 months after operation. The VAS is the most commonly used method to assess pain and consists of a 10-cm horizontal line with one end labeled as 0 cm representing “no pain” and the other end labeled as 10 cm representing “severe pain.” Patients will be instructed to make a mark on the line to indicate their pain intensity. The VAS scores range from 0–10 points, with 0 indicating no pain, 1–3 indicating mild pain, 4–6 indicating moderate pain, and 7–10 indicating severe pain
- MRI examination to evaluate cartilage repair and regeneration at baseline, 3, 6, 12, and 18 months after operation
- Adverse events, such as fever, joint pain, swelling, effusion, and rejection after cartilage transplantation, will be observed at 3, 6, 12, and 18 months postoperatively. Common adverse events after microfractures include joint fever, joint pain, joint swelling, and effusion. All outcome evaluations will be performed independently and blindly by experienced evaluators

#### Adverse events

##### Safety

Standard operating procedures for adverse events and severe adverse events will be developed to ensure that any adverse reactions during the experiment will be treated quickly to protect the participants.

#### Definitions

##### Adverse events

Adverse medical events may occur after cartilage transplantation or microfracture surgery, but they do not necessarily have a causal relationship with treatment (Table 1).

**Table 1:** Adverse event registration form

Adverse events		Appearance time	Duration
Fever	□		
Joint pain	□		
Joint snapping	□		
Joint noose	□		
Joint swelling	□		
Activity limitation	□		
Pruritus	□		

Common adverse reactions after cartilage transplantation include fever, joint pain, swelling, and effusion. Common adverse reactions after microfracture surgery include fever, joint pain, joint swelling, and effusion.

Adverse events will be relieved by nursing care, such as reducing activity, intermittent ice compress, and the use of non-steroidal anti-inflammatory analgesic drugs.

##### The severity of adverse events

Adverse events will be classified into three levels: general adverse events, vital adverse events, and severe adverse events.

##### Relationship with tissue-engineered cartilage

The correlation between adverse events and tissue-engineered cartilage will be categorized into “definitely related,” “probably related,” “possibly unrelated,” “irrelevant” or “undetermined.”

##### Severe adverse events

All events occurring during the trial requiring hospitalization or prolonged hospitalization, or resulting in disability, or affecting the ability to work, or with a risk of death or life-threatening events will be recorded.

#### Adverse event recording

All adverse events during the experiment will be collected until the end of the study.

#### Recording and reporting

All adverse events will be recorded by physicians, including description of adverse events, occurrence time, end time, severity, frequency, and treatment record.

Once a severe adverse event occurs, physicians will not only give necessary treatment, but also truthfully report to the local Food and Drug Administration Bureau and the National Food and Drug Administration Bureau within 24 h, as well as promptly report to the Ethics Committee. The data, treatment, and follow-up results will be noted in the report.

### Withdrawal criteria

Patients can withdraw from the study for any of the following reasons: severe adverse events (such as postoperative infections that are not eased within 1 week after surgery) related to the research; severe complications or rejection; the patient is asked to withdraw from the trial; considering the security or the benefit of the patient, the researchers believe that the patient is no longer suitable for continuing clinical validation; the patient fails to meet surgical requirements under arthroscopy; or is lost to follow-up. The time and cause of withdrawal will be recorded on an electronic Case Report Form (eCRF) in detail. When the study is discontinued, the patient should be assessed at the final visit except for those lost to follow-up. Free strategies for referral, physical examination, and MRI review will be conducted during the trial to improve patient compliance.

For patients who are out of the group and cannot be replaced or re-enter the clinical trial, their data will be treated as follows: (1) all source data and source files related to all withdrawn participants will be retained for retention and intent-to-treat analysis. After withdrawal, the researchers will attempt to contact the patient by telephone or mail to ask the reasons for withdrawal; (2) the time and cause of withdrawal will be recorded in the eCRF in detail; and (3) if there is judged to be a causal relationship with the trial, withdrawal due to adverse events will be recorded in the eCRF and the sponsor will be notified. For such patients, follow-up will be conducted until adverse events are resolved or stabilized.

### Data collection, management, and analysis

We will collect demographic data including the patient’s name, gender, date of birth, age, body weight, height, and Body Mass Index, will be recorded. The eCRF will be written in a unified format, clear and legible, including patient’s details, chief complaint, present illness history, past history, personal history, family history, physical examination, special examination, auxiliary examination, laboratory examination, final diagnosis, surgical records, and patient signature.

Two data clerks will input duplicate data into a computer database. The two separate sets of data will be compared by computer software, and modified according to the eCRF. Five percent of all case data will be randomly selected for manual checking. If the data error is greater than 0.15%, all the data in the database will be manually checked.

All participant data will maintain personal privacy. The full names of the subjects will not be present in records or documents. Only their abbreviated names will be displayed. Clinical trial data will include written data and electronic data. Electronic data will be stored in a specialized computer and managed by a data administrator using a password. Written data will be stored and locked in a fixed location, and the keys will be kept by an administrator.

### Data monitoring

An independent Data Monitoring Committee approved by the PLA General Hospital will be responsible for data monitoring, including protocol violations, recruitment rate, adverse events and participant compliance, and has the right to access all the trial data, but with no conflict of interest.

### Auditing

An inspector will audit the incoming data monthly and, if necessary, data queries will be raised. The inspector will review whether the eCRF is completed accurately. All discrepancies in the eCRF will be corrected by the researchers or authorized personnel in the appropriate manner.

### Sample size

Based on our previous experience [25] and pilot study results, we assumed that the therapeutic efficiency on full-thickness cartilage injury of the knee joint would be 78% for microfracture surgery and 97% for autologous, cell-derived, tissue-engineered cartilage transplantation. Considering *α* = 0.05 (two-sided), and power = 80%, the necessary sample size was calculated as *n* = 84. With a predicted dropout rate of 20%, the required sample size was calculated as *n* = 50 per group.

### Statistical analysis

Data will be analyzed using SPSS19.0 software (IBM Corp., Armonk, NY, USA). A statistical significance level of 0.05 will be used. Normally distributed data will be expressed as the mean ± standard deviation, and non-normally distributed data will be expressed as the median, minimum, and maximum. Classified variables will be expressed as a number and percentage.

The analysis will be performed on the basis of the intent-to-treat principle. A full analysis set following the principle of intent-to-treat will consist of a data set for all patients who participated in the trial, regardless of compliance or withdrawal. For patients who are lost to follow-up, the missing data will be imputed by the last-observation-carried-forward method based on the final observed value.

Descriptive statistics will be used for baseline feature data. Pearson’s chi-squared test or Fisher’s exact test will be used for intergroup comparisons of categorical variables, such as curative outcomes and postoperative incidence of adverse events. For independent variables, such as IKDC score, VAS score, T2 value, and △*R1*, an independent sample *t* test or the Mann-Whitney *U* test will be used.

A multivariate logistic regression analysis model will be used to adjust for possible confounding variables such as age, sex, etiology, cartilage defect area, meniscus injury, course of disease, severity of cartilage injury, postoperative complications, and surgeon (experience).

### Ethics and dissemination

#### Ethical approval and informed consent

The study protocol was approved by the Ethics Committee of the PLA General Hospital (approval No. S2015-084-01). This trial will be conducted in accordance with the World Medical Association Declaration of Helsinki (revised version of Seoul, 2008) and international standards of Good Clinical Practice.

Prior to participation in the clinical trial, the participant or their family members, guardians, and/or legal representatives will be informed of the details of the clinical trial, including known, foreseeable risks, and possible adverse events. Then, the informed Consent Form will be given to the participant or their legal representative.

#### Dissemination

Without the written consent of the sponsor, the researcher will not disclose the data and other information relating to the clinical trial to a third party or for any other purpose. In any case that the researchers publish or reveal their research findings from the test products in the clinical trial, written consent must be given by the sponsor. If the researcher wishes to publish the information related to the trial the study, the original manuscript will be reviewed and approved by the sponsor at 60 days prior to submission or presentation. If there exists a problem related to the scientific findings, stringency, or compliance with laws and regulations, the sponsor will discuss these issues with the researchers. The sponsor cannot modify the scientific content of the manuscript and has no right to conceal relevant information. The authors’ signatures of the articles published in this study will be based on the author’s signature guidelines, such as the *Uniform Requirements for Manuscripts Submitted to Biomedical Journals*. The original raw images, data (including computer databases), and samples obtained during the trial will be published as supplementary information in peer-reviewed academic journals and published data will be released at www.figshare.com. The results of the trial will be disseminated through peer-reviewed publications and presentations at relevant conferences.

### Protocol amendments

All protocol modifications must be signed by the sponsor and dated, and then released. The modified protocol will be approved by the Ethics Committee prior to implementation. No program deviations should happen during the study. If so, appropriate measures should be taken immediately. Causes of a program deviation and its details should be recorded in a CRF and in the original eCRF. The program deviation table and CRF will be kept in the research unit and by the sponsor, respectively.

### Provision

The patient as the insured will be indemnified in respect of postoperative complications stated in the schedule during the period of postoperative follow-up, physical examination, and rehabilitation guidance according to the medical accident insurance purchased preoperatively.

## Discussion

This study is designed to use patients from the Institute of Orthopedics of the Chinese PLA General Hospital to investigate a fourth-generation, tissue-engineering-oriented scaffold to construct tissue-engineered natural cartilage. Patients with full-thickness cartilage injury of the knee will be treated with the tissue-engineered cartilage, and improvement in the knee joint function, symptoms and pain, and adverse events will be observed. Microfractures will be used as controls for the comparative analysis. This trial will attempt to confirm whether tissue-engineered cartilage is more suitable for the clinical repair of full-thickness cartilage injury of the knee.

The characteristics of this study are: (1) a prospective, randomized controlled clinical trial, to observe the effectiveness and safety of autologous, cell-derived, tissue-engineered cartilage for the repair of full-thickness cartilage injury of the knee and (2) it is the first study to construct a completely natural, tissue-engineered cartilage using a tissue-engineering-oriented scaffold, for the clinical repair of articular cartilage injury of the knee, with a sample size of 100 cases. The trial results should have reliability and clinical feasibility.

This study also has some limitations: (1) because of the initial development of the tissue-engineering-oriented scaffold to build a completely natural, tissue-engineered cartilage, young patients with small cartilage defects (no larger than 5 cm^2^) will be enrolled, and further investigation is warranted to observe the therapeutic efficacy of the tissue-engineered cartilage on large-area cartilage defects in older people and (2) it is limited by a short-term follow-up (only 1.5 years), and therefore, long-term efficacy remains to be investigated. Therefore, the full clinical effect of a completely natural tissue-engineered cartilage needs to be studied further.

### Trial status

Currently recruiting.

## Appendix

**Table 2:** Lysholm score sheet

Lysholm score			Left knee	Right knee
Claudication (5 points)	No 5	5		
	Mild or periodically 3	3		
Need support (5 points)	No 5	5		
	Cane or crutch 2	2		
	Cannot bear 0	0		
Angina (15 points)	No 15	15		
	Snagging without pain 10	10		
	Occasional angina 6	6		
	Frequent angina 2	2		
	Joint angina 0	0		
Joint instability (25 points)	No 25	25		
	Rarely 20	20		
	Frequently in heavy exercise 15	15		
	Occasional in daily life 10	10		
	Frequent in daily life 5	5		
	Each step 0	0		
Pain (25 points)	No	25		
	Rarely	20		
	Obvious during heavy exercise	15		
	Obvious after 2 km walking	10		
	Obvious within 2 km walking	5		
	Continuous	0		
Swelling (10 points)	No	10		
	Heavy exercise	6		
	Daily exercise	2		
	Continuous	0		
Stairs (10 points)	No	10		
	Mild difficulty	6		
	One step	2		
Squatting (5 points)	No	5		
	Mild difficulty	4		
	Less than 90°	2		
	Cannot	0		
		Total		

## Additional file

Additional file 1: SPIRIT Checklist. (DOC 115 kb)

## Abbreviations

dGEMRIC: Delayed Gadolinium-enhanced Magnetic Resonance Imaging of Cartilage
eCRF: Electronic Case Report Form
FCD: Fixed charge density
GAG: Cartilage glycosaminoglycan
IKDC: International Knee Documentation Committee
VAS: Visual Analog Scale
